# Characterization of the Antigenic and Immunogenic Properties of the Gametocyte Antigen 56 from *Eimeria necatrix*

**DOI:** 10.3390/ani15121750

**Published:** 2025-06-13

**Authors:** Feiyan Wang, Liqin Cao, Lele Wang, Jinjun Xu, Jianping Tao, Dandan Liu

**Affiliations:** 1 College of Veterinary Medicine, Yangzhou University, Yangzhou 225009, China; dx120210181@stu.yzu.edu.cn (F.W.); 18252736323@163.com (L.W.); jjxu@yzu.edu.cn (J.X.); yzjptao@126.com (J.T.); 2 Jiangsu Co-Innovation Center for Prevention and Control of Important Animal Infectious Diseases and Zoonoses, Yangzhou University, Yangzhou 225009, China; 3 Joint International Research Laboratory of Agriculture and Agri-Product Safety, The Ministry of Education of China, Yangzhou University, Yangzhou 225009, China; 4 Jiu Long Animal Husbandry and Veterinary Station, Taizhou 225300, China; 18762321667@163.com

**Keywords:** *Eimeria necatrix*, gametocyte antigens, recombinant subunit vaccine, Immunoprotection

## Abstract

Coccidiosis, caused by *Eimeria* spp., significantly reduces poultry productivity and causes major economic losses. Traditional control methods are limited by drug resistance and high production costs. Recent genomic and bioinformatic advances have enabled the identification of novel antigens, making recombinant subunit vaccines a promising next-generation strategy by eliciting robust cellular and humoral immune responses. This study investigates the *E. necatrix* gametocyte protein 56 (EnGAM56) as a potential candidate for recombinant subunit vaccines. The full-length *E. necatrix* gametocyte *gam56* gene (En*gam56*-F) was amplified, expressed in vitro, and characterized via SDS-PAGE and Western blot. Immunofluorescence assays revealed that EnGAM56-F is specifically localized in gametocytes and unsporulated oocysts. Chickens immunized with recombinant proteins (rEnGAM56-F and rEnGAM56-T) were evaluated for immunoprotection against *E. necatrix* infection through lesion scores, weight gain, oocyst production, anticoccidial index (ACI), and antibody and cytokine levels. The synergistic effects were evaluated by employing various combinations of recombinant proteins, including rEtGAM22, rEtGAM56-T, and rEtGAM59. Results showed that EnGAM56-F encodes a 468-amino acid protein with distinct tyrosine-serine-rich and proline-methionine-rich regions. rEnGAM56-F was specifically recognized by both anti-6 × His tag antibodies and convalescent serum from chickens infected with *E. necatrix*. Both rEnGAM56-F and rEnGAM56-T provided immune protection, with rEnGAM56-T showing superior efficacy. The combination of rEnGAM (22 + 59 + 56-T) yielded the strongest immune response, followed by rEnGAM (22 + 56-T). These findings highlight the potential of EnGAM56 as a candidate for recombinant subunit anticoccidial vaccines.

## Simple Summary

Coccidiosis is a major disease in poultry, leading to reduced productivity and economic losses. Current control methods face limitations due to drug resistance. In this study, we evaluated a gametocyte protein 56 from *Eimeria necatrix* (EnGAM56) as a new vaccine candidate. The gene was cloned, expressed, and tested for its immune protection in chickens. Immunization with recombinant protein rEnGAM56 reduced disease severity and improved immune response. Notably, combining rEnGAM56 with other proteins enhanced protection. These results support the development of recombinant subunit vaccines as a safer and more effective strategy against coccidiosis.

## 1. Introduction

Coccidiosis is a potentially debilitating disease caused by the *Eimeria* parasite, characterized by impaired growth performance and decreased feed conversion rates [1]. It is estimated that the global economic burden of coccidiosis exceeded £ 12.9 billion in 2022 [2]. Among the various *Eimeria* species, *Eimeria necatrix* is identified as a highly pathogenic protozoan, primarily targeting the intestinal tract of chickens and inducing acute intestinal coccidiosis in poultry [3]. Currently, the primary strategies for controlling chicken coccidiosis rely on chemoprophylaxis and the administration of live attenuated vaccines. However, limitations in vaccine efficacy, the emergence of drug-resistant *Eimeria* strains, and increasing regulatory restrictions on antibiotic usage have prompted a shift in control strategies [4]. In this context, subunit vaccines derived from parasite-specific antigens or recombinant proteins expressed from cloned DNA represent a promising alternative to overcome these challenges [5].

Immunoprotective genes from the *Eimeria* genome have been identified and selected for cloning and expression, facilitating the development of highly immunogenic subunit vaccine candidates [6]. To date, CoxAbic^®^, the only commercially available subunit vaccine against chicken coccidiosis, comprises three major gametocyte-derived antigens from *Eimeria maxima*: 230 kDa, 82 kDa, and 56 kDa proteins. Field trials have demonstrated that CoxAbic^®^ reduces oocyst shedding by approximately 50–80% [7]. Inspired by these findings, researchers have focused on identifying additional gametocyte antigens, such as GAM22 [8,9,10], GAM56 [10,11,12,13], and GAM59 [14], which may further enhance vaccine efficacy.

*Gam22* is the first identified multi-copy gene within *Eimeria* species, characterized by an exceptionally high copy number and highly conserved sequences among its copies [8]. EtGAM22 is localized to wall-forming bodies type 2 (WFB2), where it is involved in the formation of the inner oocyst wall and/or the Stieda body in *Eimeria tenella* [10]. In contrast, EnGAM22 is distributed within wall-forming bodies type 1 (WFB1) and plays a role in the formation of the outer oocyst wall in *E. necatrix* [15]. The *Gam56* gene is a single-copy, intronless gene initially identified in the gametocytes of *E. maxima* [11] and encodes the EmGAM56, which localizes to both WFB2 and the inner oocyst wall in *E. maxima*. The *Gam59* gene (also referred to as Et*gam56* tmp2), encoding a GAM56-like protein (GAM59), was first identified in the *E. tenella* genome and is positioned adjacent to the *gam56* cDNA (Et*gam56* tmp1) [10]. In *E. necatrix*, EnGAM59 similarly localizes to WFB2 and contributes to the formation of the inner oocyst wall [15]. Previous studies in our group demonstrated that En*gam22* and En*gam59* are promising candidates for the development of recombinant subunit vaccines against coccidiosis [9,14]. Further investigation of the *gam56* gene is expected to yield valuable insights into the design of more effective vaccination strategies for the prevention and control of coccidiosis.

In this study, we successfully cloned and expressed the full-length En*gam56* gene (En*gam56*-F) and subsequently evaluated its potential, along with that of a truncated version (En*gam56*-T), as candidate subunit immunogens for protection against *E. necatrix* infection. In addition, the protective efficacy of immunization with individual recombinant proteins (rEnGAM22, rEnGAM56, and rEnGAM59) and their combination was assessed in chickens.

## 2. Materials and Methods

### 2.1. Protein, Animals, and Parasites

Recombinant gametocyte proteins of *E. necatrix*-rEnGAM22, rEnGAM59, and the truncated rEnGAM56-T-were successfully expressed in our laboratory using a prokaryotic expression system [9,14]. rEnGAM22 contains a region rich in histidine and proline [9], while rEnGAM59 protein contains both a tyrosine-serine-rich region and a proline-methionine-rich region [14]. rEnGAM56-T, a truncated form of the rEnGAM56-F protein, retains a domain enriched in tyrosine and serine [9]. Preliminary evaluations have shown that both rEnGAM22 and rEnGAM59 are immunogenic and confer protective immunity against *E. necatrix* infection.

One-day-old yellow-feathered broiler chickens were purchased from Jiangsu Jinghai Poultry Industry Group Co., Ltd. (Nantong, Jiangsu, China), reared in a coccidian-free environment, and provided with water and food *ad libitum*.

The *E. necatrix* Yangzhou strain was previously isolated and is routinely maintained in our laboratory. A total of 20 chickens, aged 15 days, were orally inoculated with 20,000 sporulated oocysts. Feces samples were collected daily from 7 to 12 days post-infection (PI). Unsporulated oocysts were isolated and purified using a saturated salt solution floatation method, as described in previous reports to allow for sporulation [16]. The resulting sporulated oocysts were then stored at 4 °C in 2.5% (*w*/*v*) potassium dichromate until further use. Gametocytes were isolated and purified from experimentally infected chickens using previously published methods [15]. Briefly, mucosal tissues were scraped from the caecum of chickens infected with 30,000 sporulated oocysts, and the tissues were digested in SAC buffer (1 mM phenylmethanesulfonyl fluoride (PMSF), 1 mg/mL bovine serum albumin, 170 mM NaCl, 10 mM Tris-HCl (pH 7.0), 10 mM glucose, and 5 mM CaCl_2_) containing 0.5 mg/mL hyaluronidase. The digested material was sequentially filtered through 17 μm and 10 μm polymer meshes, with gametocytes retained on the mesh being washed with cold SAC, collected by centrifugation at 1000× *g* for 5 min, and stored at −80 °C until further use.

### 2.2. Plasmid Construction

The total RNA of gametocytes was extracted and transcribed into cDNA using an RNA Extraction Kit (TaKaRa, Tokyo, Japan) and Reverse Transcriptase Kit (TaKaRa), following the manufacturer’s instructions. As the amplicons were expected to have high GC contents, the full-length *gam56* gene (En*gam56*-F) was amplified in segments using RT-PCR with PrimeSTAR^®^ GXL DNA Polymerase (TaKaRa). Three overlapping fragments were amplified with three pairs of primers designed based on the *gam56* sequence of the *E. necatrix* Houghton strain (GenBank accession number: XM_013578447.1) (Table 1). The PCR cycling conditions were as follows: 98 °C for 10 s, 58 °C for 30 s, and 68 °C for 1.5 min.

The fragments were subsequently subcloned into pGEM^®^-T-easy vector (Promega, Madison, WI, USA) and sequenced by Beijing Genomics Institute (BGI). The obtained sequences were spliced and analyzed using the DNAMAN 6.0 software. After removing the signal peptides (first 20 amino acids), the En*gam56*-F gene was further optimized for prokaryotic expression and synthesized by Genscript (Nanjing, China).

Signal peptide prediction was obtained by the SignalP 5.0 server https://services.healthtech.dtu.dk/services/SignalP-5.0/ (accessed on 12 June 2025). Antigenic peptides were predicted by using the methods of Kolaskar and Tongaonkar http://imed.med.ucm.es/Tools/antigenic.pl (accessed on 12 June 2025). Amino acid alignments were performed with the ClustalW algorithm in DNAMAN 7.0 software https://www.lynnon.com/ (accessed on 12 June 2025). Protein structure prediction was identified through the SOPMA https://npsa-pbil.ibcp.fr/cgi-bin/npsa_automat.pl?page=npsa_sopma.html (accessed on 12 June 2025), and domain analysis was carried out with InterPro http://www.ebi.ac.uk/interpro/ (accessed on 12 June 2025).

### 2.3. Expression and Purification of Recombinant Proteins

Recombinant expressing bacteria pET28a(+)-En*gam56*-F/BL21 was cultured in LB medium at 37 °C to OD600 = 0.8, and a final concentration of 1.0 mM isopropyl β-D-1-thiogalactopyranoside (IPTG; Promega Corp., Madison, WI, USA) was added to induce recombinant protein (rEnGAM56-F) production. The rEnGAM56-F was purified using a Nickel-Nitrilotriacetic Acid (Ni-NTA) affinity chromatography column (GenScript, Nanjing, China) according to our previous study [9], analyzed by 12% SDS-PAGE with Coomassie Brilliant Blue G-250 staining (Sigma-Aldrich, St Louis, MO, USA), and treated with the ToxinEraser endotoxin removal kit (Genscript).

### 2.4. Preparation of Immunoglobulin Serum

Mouse anti-rEnGAM56-F polyclonal antibody (anti-rEnGAM56-F pAb) was generated by immunizing six 6-week-old female BALB/c mice with rEnGAM56-F. Each mouse was intramuscularly injected with rEnGAM56-F (20 μg of protein in 50 μL of PBS), emulsified with 50 μL of QuickAntibody-Mouse3W adjuvant (Biodragon, Beijing, China). Booster immunizations were administered 14 days later, and blood was collected to isolate serum 21 days later.

Convalescent serum of chicken with *E. necatrix* infection had already been prepared and stored at −80 °C in our lab.

### 2.5. Western Blot Analysis

Total protein was extracted from gametocytes placed in 1.5 mL RNase-free microcentrifuge tubes containing 200 μL cell lysis buffer (Xinsaimei, Shanghai, China) and 2 μL protease inhibitor (Xinsaimei). The samples were incubated on ice for 30 min, followed by ultrasonic disruption in an ice bath for 5 min (30% power, 2 s on/3 s off cycles). The lysates were then centrifuged at 10,000× *g* for 10 min at 4 °C, and the resulting supernatants containing total protein were collected for further analysis.

The identification of EnGAM56-F protein and rEnGAM56-F was performed using the Wes Capillary Western Blot System (ProteinSimple, San Jose, CA, USA), with a 12–230 kDa Separation Module (#SM-W003). The antigen specificity of rEnGAM56-F was also analyzed using this system. Briefly, the mouse anti-6 × HIS tag monoclonal antibody (1:100 dilution, BIO BASIC, Markham, Canada), mouse negative serum (1:50 dilution), mouse anti-rEnGAM56-F pAb (1:50 dilution), chicken negative serum (1:20 dilution), and the convalescent serum of chicken infected with *E. necatrix* (1:20 dilution) were used as primary antibodies, respectively. The HRP-labeled rabbit anti-mouse IgG (1:1000 dilution, BIO BASIC) and rabbit anti-chicken IgG (1:1000 dilution, BIO BASIC) were used as secondary antibodies. All antibodies were diluted in antibody diluent (ProteinSimple). Positive signals were visualized using Compass for SW software (V4.0.0, Protein Simple).

### 2.6. Indirect Immunofluorescence Localization

The rabbit anti-rEnGAM59 pAb specifically localized EnGAM59 to the WFB2 in gametocytes, serving as a marker to differentiate between WFB1 and WFB2 [15].

Freshly isolated gametocytes and unsporulated oocysts were evenly spread onto glass slides and fixed with methanol at −20 °C for 10 min. The samples were then permeabilized with 0.1% Triton X-100 (Beyotime, Shanghai, China) for 10 min at room temperature. After being blocked with 3% BSA in PBS for 3 h at 37 °C, the slides were incubated overnight at 4 °C with anti-rEnGAM56-F pAb (1:200 dilution) or rabbit anti-rEnGAM59 pAb (1:100 dilution, prepared in our laboratory). The slides were washed three times with 0.03% Tween-20 in PBS (PBST) for 15 min each and then incubated for 1 h at 37 °C with either fluorescein isothiocyanate (FITC)-conjugated goat anti-rabbit IgG (1:100 dilution; MultiSciences, Hangzhou, China) or Cy3-conjugated goat anti-mouse IgG (1:100 dilution; MultiSciences).

Under the fluorescence microscope (Leica DM2500, Leica Microsystems GmbH, Wetzlar, Germany), sealing tables with anti-fluorescence quenching agents (Roche, Basel, Switzerland) were examined.

### 2.7. Vaccinations and Challenge Experiment

Two immunization trials were conducted. The first trial aimed to evaluate the protective efficacy of rEnGAM56-F and rEnGAM56-T, as well as to determine the optimal immunization dosage. The second trial was designed to assess the protective efficacy of a combined immunization strategy involving rEnGAM56, rEnGAM22, and rEnGAM59.

For immune dose screening, doses of 50 μg, 100 μg, and 200 μg were selected based on previous findings from our laboratory to evaluate the immune protective efficacy of different gametocyte proteins [14,17]. Accordingly, chickens immunized with rEnGAM56-F and rEnGAM56-T received doses of 50 μg, 100 μg, and 200 μg, respectively. For rEnGAM22, prior studies demonstrated that a 50 μg dose provided optimal immune protection [9], while a 200 μg dose was most effective for rEnGAM59 [14]. In the present study, the immunization dose of rEnGAM59 was reduced to 100 μg per chicken to minimize protein load, ensuring safety and preventing potential overstimulation of the immune system.

**Figure 1:**
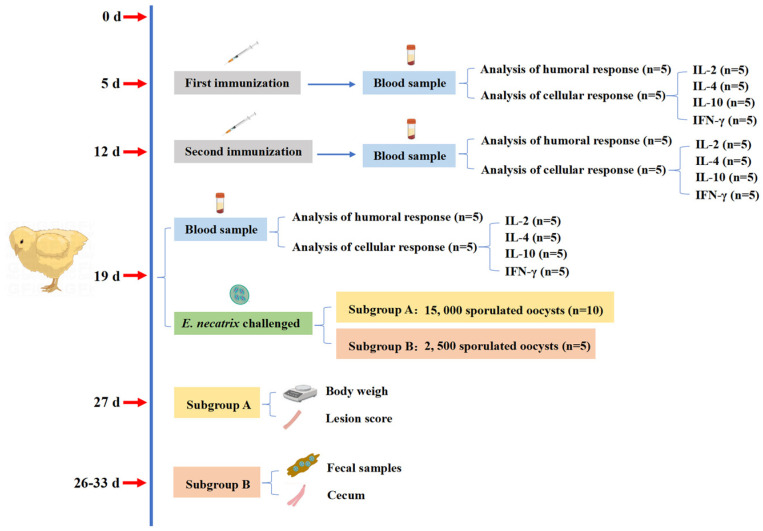
The immunization procedure and regimen of chickens.

The experimental design and its immunization procedure were shown in Figure 1. Chickens aged 5 days within a similar weight range were randomly divided into eight groups, each consisting of 15 chickens. Experimental groups contained immunized groups (IC): rEnGAM56-F immunized groups (rEnGAM56-F-H: 200 μg per chicken, rEnGAM56-F-M: 100 μg per chicken, and rEnGAM56-F-L: 50 μg per chicken) and rEnGAM56-T immunized groups (rEnGAM56-T-H: 200 μg per chicken, rEnGAM56-T-M: 100 μg per chicken, and rEnGAM56-T-L: 50 μg per chicken). Control groups included the unimmunized and challenged group (UC) and the unimmunized and unchallenged group (UU).

In a combined protocol, the rEnGAM22, rEnGAM56-T, and rEnGAM59 were combined to immunize chickens in different combinations. The immunized groups consisted of three single-immunized groups, namely rEnGAM22 (50 μg per chicken), rEnGAM56-T (50 μg per chicken), and rEnGAM59 (100 μg per chicken), as well as two co-immunization groups: One co-immunized with rEnGAM22 (50 μg per chicken) and rEnGAM56-T (50 μg per chicken), and the other co-immunized with rEnGAM22 (50 μg per chicken), rEnGAM56-T (50 μg per chicken), and rEnGAM59 (100 μg per chicken). Control groups included an unimmunized and challenged group (UC) and an unimmunized and unchallenged group (UU). Immunizations were administered via subcutaneous injection at one-week intervals. Complete Freund’s adjuvant (Sigma-Aldrich) was used for the first immunization, while incomplete Freund’s adjuvant (Sigma-Aldrich) was utilized for the second immunization. Recombinant protein was diluted in PBS and mixed with the corresponding adjuvant at a 1:1 volume ratio.

To evaluate vaccine efficacy, a low-dose vaccination strategy was employed to better simulate natural infection conditions and enable a more accurate assessment of immune protection. Seven days after the second immunization (day 19, D19), each group was randomly divided into two subgroups. Subgroup A (*n* = 10) was orally challenged with 15,000 sporulated oocysts, while subgroup B (*n* = 5) received 2,500 sporulated oocysts.

For subgroup A, the protective efficacy of the treatments was evaluated based on survival rate, body weight gain (BWG), relative weight gain (RWG), and lesion score. The survival rate (%) was calculated with the formula (number surviving per group/total number of each group) × 100. BWG1 was calculated for days 5–19 in the immunization period, BWG2 was calculated for days 19–27 in the challenge period, and RWG (%) was calculated with the formula BWG of IC or UC groups/BWG of UU group × 100. Lesion scores were assessed on day 8 PI (27 days of age) using a 0 to 4 scale, as described by Johnson and Reid [18]. Two independent observers assessed the extent of intestinal bleeding, evaluated changes in the intestinal wall, and examined the condition of intestinal contents, assigning lesion scores accordingly. For subgroup B, the protective efficacy was evaluated based on oocyst reduction (OR). The OR (%) was calculated with the formula (OR of UC group—OR of IC groups)/OR of UC group) × 100. The oocyst shedding was measured between days 7 and 14 PI (26–33 days of age) using the McMaster counting method, as previously described [19].

The anticoccidial index (ACI) was used to evaluate the anticoccidial efficacy. The ACI is calculated as ACI = (survival rate + RWG) × 100 − (lesion value + oocyst value). The lesion value is lesion score × 10. The oocyst value is assigned based on the oocyst ratio: 0 if the oocyst ratio is 0–1%, 5 if 1–25%, 10 if 26–50%, 20 if 51–75%, and 40 if 76–100%, where the oocyst ratio is defined as the oocyst outputs of IC groups divided by that of UC groups. The ACI values above 180 were deemed excellent, 160–180 as marked, 140–160 as moderate, 120–140 was classified as mild or slight, and below 120 as inefficacy [19].

### 2.8. Detection of Serum Antibody Levels

Blood samples were collected from five randomly selected chickens in each group at three time points: Prior to the first immunization (Day 5, D5), 7 days after the primary immunization (Day 12, D12), and 7 days after the secondary immunization (Day 19, D19). The samples were kept at room temperature for 2 h, then centrifuged at 2500 g for 10 min. The resulting serum was stored at −80 °C for subsequent analysis.

Indirect enzyme-linked immunosorbent assays (ELISAs) were employed to detect serum antibody levels of the chickens. Briefly, 96-well plates were coated overnight at 4 °C with 1 μg/well of purified rEnGAM22, rEnGAM59, rEnGAM56-F, or rEnGAM56-T. For the combined immunization group, rEtGAM22 and rEtGAM56 were mixed at a 1:1 ratio and coated, and rEtGAM22, rEtGAM56, and rEtGAM59 were mixed at a 1:1:1 ratio before coating. Plates were washed three times with 0.05% Tween-20 in PBS (PBST) for 15 min and then blocked with 1.0% bovine serum albumin (BSA; Sigma-Aldrich) in PBS for 1.5 h at 37 °C. After blocking, diluted serum samples (1:200) were added to the wells and incubated at 37 °C for 1.5 h. Plates were then washed three times with PBST, and bound antibodies were detected using horseradish peroxidase (HRP)-conjugated goat anti-chicken IgY (H + L) antibody (1:20,000; GenScript). The optical density at 450 nm (OD_450_) was measured using an ELISA reader (Sunrise-Basic, Tecan Trading AG, Männedorf, Switzerland). All samples were assayed in triplicate.

### 2.9. Determination of Serum Cytokine Levels

The cytokines interleukin-2 (IL-2), interleukin-4 (IL-4), interleukin-10 (IL-10), and interferon-γ (IFN-γ) were quantified in serum samples collected from five chickens per group on D5, D12, and D19. Quantification was performed using commercial cytokine detection kits (MEIMIAN Bio, Yancheng, China) following the manufacturer’s protocols. All serum samples were analyzed in triplicate.

### 2.10. Statistical Analysis

All data were expressed as means ± SEM, and statistical analysis was performed using the GraphPad Prism (GraphPad Prism 8.0, USA). ANOVA was used to test if there are statistically significant differences between treated and control groups. Significant differences were referred to as *: *p* < 0.05, **: *p* < 0.01, ***: *p* < 0.001, and ****: *p* < 0.0001.

## 3. Results

### 3.1. Sequence Characterization of the Full-Length Engam56 Gene

Sequences amplified by the three pairs of primers were assembled into the full-length En*gam56* gene using DNAMAN 6.0 software. The En*gam56*-F gene spans 1407 base pairs (GenBank accession: MK581051) and consists of a full open reading frame that encodes 468 amino acids. The first 20 amino acids constitute the signal peptide, and the predicted molecular weight of the EnGAM56-F is approximately 55.0 kDa.

**Figure A1:**
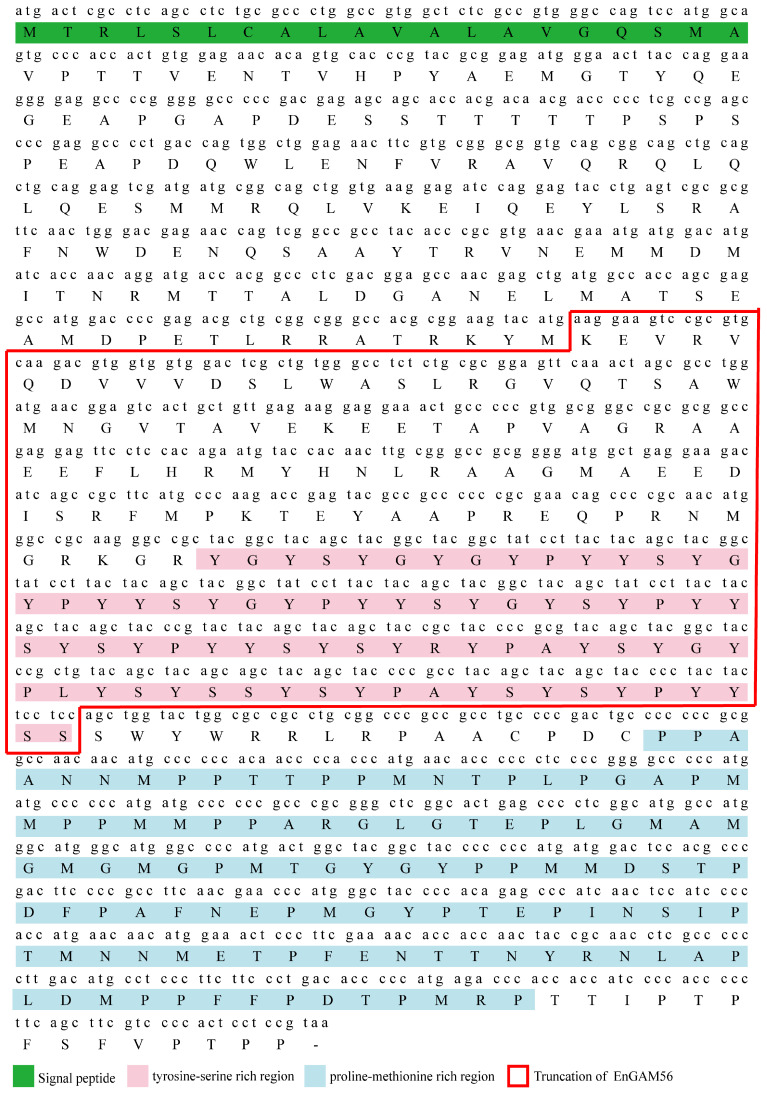
Amino acid sequence of EnGAM56-F and EnGAM56-T (inside the red box). Boxes with different colors and positions represented different structural motifs. The EnGAM56-F contains a signal peptide (1-20 aa, green), a tyrosine-serine-rich region (246-323 aa, pink), and a proline-methionine-rich region (338-454 aa, light green). The EnGAM56-T contains a tyrosine-serine-rich region (91-167 aa, pink).

Analysis of amino acid composition revealed that the En*gam56*-F gene contains 33 strongly basic amino acids (K, R), 44 strongly acidic amino acids (D, E), 106 hydrophobic amino acids (A, I, L, F, W, V), and 160 polar amino acids (N, C, Q, S, T, Y). Furthermore, the protein sequence is rich in proline (12.6%), tyrosine (10.9%), serine (7.7%), and methionine (7.5%), respectively. The antigenicity analysis showed that the EnGAM56-F protein contained a tyrosine- and serine-enrichment region (246–323 aa) and a proline- and methionine-rich region (338–454 aa) (Figure A1).

**Figure A2:**
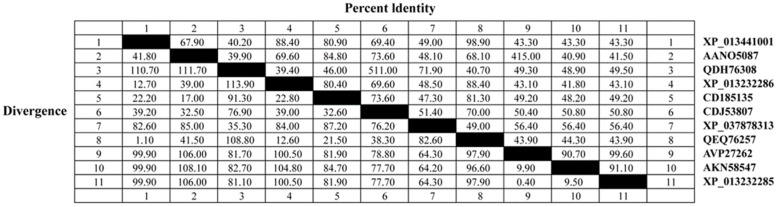
The sequence homology among various gametocyte proteins was analyzed. EnGAM56-F exhibited the highest homology to the Houghton strain EnGAM56, with 98.90% identity (XP_013441001).

A multiple amino acid alignment of EnGAM56-F and other gametocyte proteins revealed that EnGAM56-F (Accession: QEQ76257.1) has 98.90% identity to Houghton strain EnGAM56 (XP_013441001), 97.90% identity to EtGAM59 (AVP27262) and EtGAM56-like (XP_013232285), 96.60% identity to EnGAM59 (AKN58547), 88.40% identity to EtGAM56 (XP_013232286), and 81.30% and 70.00% identity to EpGAM56 (CDI85135) and EbGAM56 (CDJ53807) (Figure A2).

### 3.2. Expression, Purification, and Immunoblotting Analysis of Recombinant Protein

**Figure 2:**
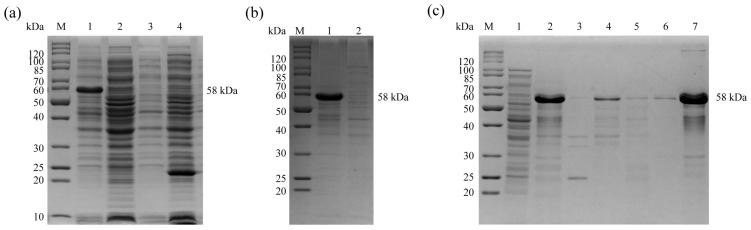
Expression and purification of rEnGAM56-F. (**a**) Expression of the rEnGAM56-F with IPTG induction. Lane M: Molecular weight marker; Lane 1: pET-28a(+)-Engam56-F/BL21 induced by IPTG; Lane 2: pET-28a(+)-Engam56-F/BL21 uninduced by IPTG; Lane 3: pET-28a(+)/BL21 IPTG induced; Lane 4: BL21 IPTG induced. (**b**) The solubility analysis of fusion proteins. Lane M: Molecular weight marker; Lane 1: Sediments of bacterial sonicates; Lane 2: Supernatant of bacterial sonicates. (**c**) Purified recombinant proteins resulted from Ni-NTA affinity chromatography purification. Lane M: Molecular weight marker; Lane 1: Supernatant of bacterial sonication; Lane 2: Sediments of inclusion bodies after solubilizing by urea; Lane 3: Supernatant of inclusion bodies after solubilizing by urea; Lane 4: Effluent after binding with Ni-NTA; Lane 5: Washing buffer for the last time; Lane 6: Elution buffer for the first time; Lane 7: Elution protein of Ni-NTA.

The pET28a (+)-En*gam56*-F was transformed into E. coli BL21 (DE3) and induced by 1.0 mM IPTG for 4 h at 37 °C. (Figure 2a, lane 1). SDS-PAGE analysis showed that the rEnGAM56-F was 58.0 kDa in size and was expressed as inclusion bodies (Figure 2b, lane 1). The rEnGAM56-F was purified by Ni-NTA affinity chromatography (Figure 2c).

**Figure 3:**
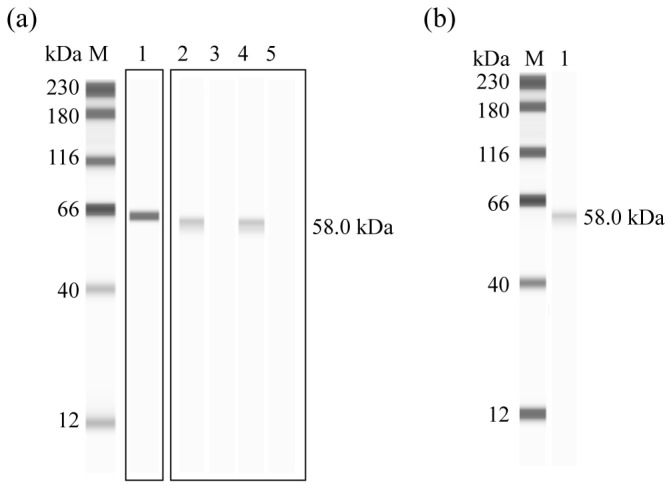
Western blot analysis of rEGAM56-F. (**a**): rEGAM56-F recognized by anti-6 × HIS monoclonal antibody (Line 1), anti-rEnGAM56-F pAb (Line 2), and convalescent serum of chicken infected with *E. necatrix* (Line 4). Lane M: Molecular weight marker; Lane 3 and 5: Negative serum from mouse and chicken, respectively. (**b**): Native gametocyte protein recognized by anti-rEnGAM56-F pAb (line 1). Lane M: Molecular weight marker.

Western blot analysis confirmed the successful expression of rEnGAM56-F, which was specifically recognized by the anti-6 × HIS monoclonal antibody (Figure 3a, lane 1), anti-rEnGAM56-F polyclonal antibody (Figure 3a, lane 2), and convalescent serum from chickens infected with *E. necatrix* (Figure 3a, lane 4). No signal was observed with negative control serum (Figure 3a, lanes 3 and 5). Moreover, the mouse anti-rEnGAM56-F pAb recognized a ~58.0 kDa protein in gametocyte extracts (Figure 3b, lane 1).

### 3.3. Immunofluorescence Localization

**Figure 4:**
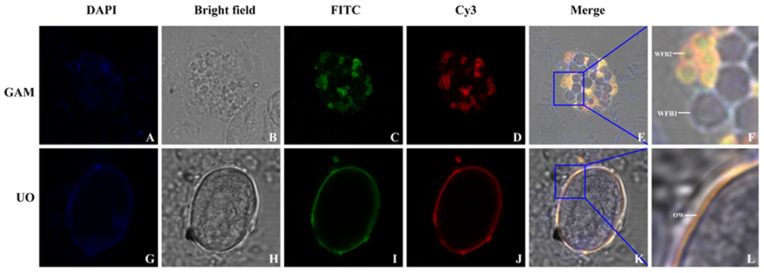
Localization of EnGAM56-F in the gametocyte (GAM) and unsporulated oocyst (UO) with anti-rEnGAM56-F pAb. (**A**,**G**): Counter-stained with DAPI; (**B**,**H**): Bright-field microscopy photographs; (**C**,**I**): Immunofluorescence localization with FITC-conjugated mouse anti-rEnGAM56-F pAb; (**D**,**J**): Immunofluorescence localization with Cy3-conjugated rabbit anti-rEnGAN59 pAb; (**E**,**K**): The superposition of different fluorescences (Merge of images). Scale bar = 5.0 μm. (**F**): Local enlarged drawing of **E**; (**L**): Local enlarged drawing of **K**.

Immunofluorescence localization analysis of EnGAM56-F in gametocytes and unsporulated oocysts was performed with mouse anti-rEnGAM56-F pAb. The results revealed that EnGAM56-F was localized not only in the wall-forming bodies (WFBs) of gametocytes but also in the oocyst wall of unsporulated oocysts (Figure 4). Previous research has identified that rEnGAM59 is predominantly localized to WFB2 (15). In this study, co-localization analysis using the rabbit anti-rEnGAM59 pAb revealed that EnGAM56-F, similar to EnGAM59, is primarily distributed on WFB2 within the gametocytes.

### 3.4. The Protective Effect of rEnGAM56

#### 3.4.1. ACI (Anticoccidial Index)

The protective efficacy of the rEnGAM56-F and rEnGAM56-T against *E. necatrix* infection was evaluated in this study (Table 2). There was no incident reported during any of the experiments, and all animals survived the challenge procedure. The BWG of chickens immunized with rEnGAM56-T and rEnGAM56-F proteins was higher than that of the UC group. Similarly, chickens vaccinated with either rEnGAM56-T or rEnGAM56-F showed a significant reduction in oocyst output and lower lesion scores compared to the UC group. In the comparison between the groups rEnGAM56-T and rEnGAM56-F, the highest RWG was observed in group rEnGAM56-T-L (84.09%), and the lowest lesion scores were recorded in group rEnGAM56-T-M (1.30 ± 1.09), which was significantly lower than those in group UC (*p* < 0.05). The OR in group rEnGAM56-T was lower than that in group rEnGAM56-F when exposed to the same challenging dose. Among all groups, the group rEnGAM56-T-L achieved the highest OR (66.20%), followed by the group rEnGAM56-T-M (58.20%), whereas the lowest OR was recorded in the group rEnGAM56-F-M (18.59%). In terms of ACI, group rEnGAM56-T-L (ACI = 158.59) had the highest value, closely followed by the groups rEnGAM56-T-M (ACI = 152.76) and rEnGAM56-F-L (ACI = 146.47), all of which were in proximity to the medium effective anticoccidial level.

#### 3.4.2. Evaluation of Serum Antibody Level

**Figure 5:**
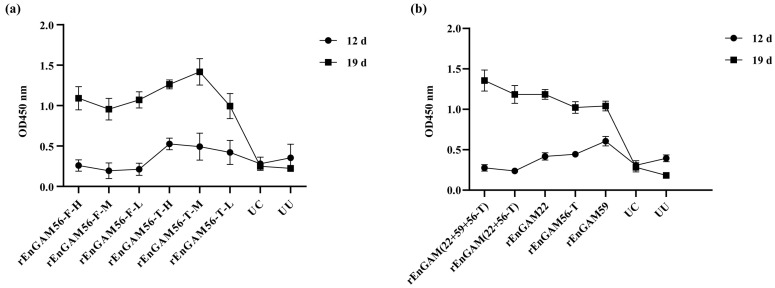
The levels of IgY after vaccination in chicken sera. (**a**) ELISA detection of IgY levels response to immunization with rEnGAM56-F or rEnGAM56-T protein. The antibody levels increased obviously in the immunized groups after the second immunization (19 days old). The highest IgY levels were found in the Group rEnGAM56-T-M, which was significantly higher than in all other groups (*p* < 0.0001). (**b**) ELISA detection of IgY levels response to immunization with single-protein or multi-protein. The Group rEnGAM (22 + 59 + 56-T) had the highest IgY levels among all groups, followed by Group rEnGAM (22 + 56-T) after the second immunization. Each bar represents the mean ± SEM (*n* = 5).

The IgY levels in each experimental group were similar on D5 and gradually increased following primary immunization, as shown in Figure 5a. On D12, the IgY levels in the groups rEnGAM56-T were significantly higher than those in the groups rEnGAM56-F (*p* < 0.05). By D19, the IgY levels in all immunized groups showed a further significant increase, with levels significantly higher than those in the groups UC and UU (*p* < 0.0001). Furthermore, the IgY levels in the groups rEnGAM56-T remained consistently higher than those in the groups rEnGAM56-F throughout the immunization program. Notably, the group rEnGAM56-T-M (OD450 = 1.42 ± 0.09) exhibited the most significant increase on D19, with responses markedly higher than those of all other groups (*p* < 0.0001), followed by the group rEnGAM56-T-H (OD450 = 1.26 ± 0.03), which showed the second highest antibody level.

#### 3.4.3. Evaluation of Serum Cytokine Levels

**Figure 6:**
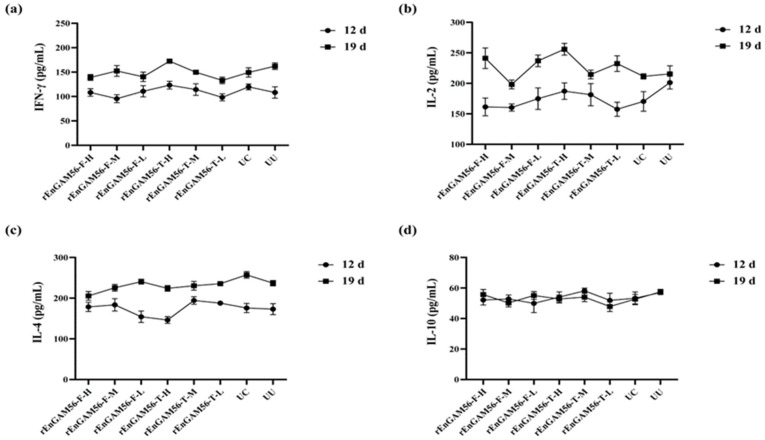
The serum cytokine level responses to rEnGAM56-F or rEnGAM56-T immunization. IFN-γ (**a**), IL-2 (**b**), IL-4 (**c**), and IL-10 (**d**) were detected by ELISA at 7 days following primary and secondary immunization. After the second immunization, no statistically significant differences were observed among the groups. However, the levels of IFN-γ and IL-2 showed an upward trend across all groups. IL-4 levels increased with the age of the chickens in all groups, while IL-10 levels remained relatively stable throughout the time points. Each bar represents the mean ± SEM (*n* = 5).

The results of serum cytokine levels (SCL) are shown in Figure 6. The levels of IFN-γ (Figure 6a), IL-2 (Figure 6b), IL-4 (Figure 6c), and IL-10 (Figure 6d) in each experimental group were similar on the D5. They started to slowly rise after the primary immunization (D12), except for IL-10. The highest level of IFN-γ was found in group rEnGAM56-T-H (172.45 ± 1.34 pg/mL) on D19. Serum IL-2 levels were elevated in the group rEnGAM56-T-H (255.95 ± 12.56 pg/mL) on D19 as compared to all other groups. The IL-4 levels increased with the age of the chickens in all experimental groups, but no significant differences were observed on D19. In terms of IL-10 levels, there were no significant differences between groups (*p* > 0.05). However, due to the antagonistic effects between Th1 (IFN-γ and IL-2) and Th2 (IL-4 and IL-10), the rEnGAM56-T-H group exhibited relatively higher levels of IFN-γ and IL-2, while IL-4 levels showed a tendency to be the lowest.

Although cytokine analysis showed no significant changes in trends between recombinant protein immunization groups and the control group, the IgY levels in the recombinant protein immunization groups were significantly increased. This suggested that the immune response was primarily mediated through the humoral immune pathway. While the IgY levels in the group rEnGAM56-T-L were lower, the highest ACI index indicates that this group may provide superior immune protection and enhanced resistance to challenges. Therefore, the rEnGAM56-T-L group was ultimately selected as the optimal immunization group.

### 3.5. The Protective Effect of Multi-Antigen Combinations

#### 3.5.1. ACI (Anticoccidial Index)

In the multi-antigen immunization experiment (Table 3). There was no incident reported during any of the experiments, and all animals survived the challenge procedure. The RWG of the group rEnGAM (22 + 59 + 56-T) (96.16%) was significantly higher than that of group UC (*p* < 0.05), followed closely by the group rEnGAM (22 + 56-T) (91.56%). Lesion scores in the immunized groups were significantly lower than in group UC (*p* < 0.05), with the group rEnGAM (22 + 59 + 56-T) showing the lowest lesion score (0.30 ± 0.08). Oocyst production was counted after the chicken began excreting oocysts, revealing that the groups rEnGAM22 (40.38%) and rEnGAM (22 + 56-T) (35.04%) displayed a reduction in oocyst shedding compared to all other groups. Additionally, the ACI for the group rEnGAM (22 + 59 + 56-T) (173.16), group rEnGAM (22 + 56-T) (161.56), and group rEnGAM22 (162.00) reached a medium anticoccidial level, indicating their superior anticoccidial efficacy compared to the other groups.

#### 3.5.2. Evaluation of Serum Antibody Level

For IgY levels (Figure 5b), there was no significant variation among all groups before the first immunization (D5). However, by D19, the IgY levels in the immunized groups were significantly higher compared to those in the groups UC and UU (*p* < 0.05). The group rEnGAM (22 + 59 + 56-T) (OD450 = 1.35 ± 0.05) had the highest IgY levels among all groups, followed by group rEnGAM (22 + 56-T) (OD450 = 1.18 ± 0.02).

#### 3.5.3. Evaluation of Serum Cytokine Levels

**Figure 7:**
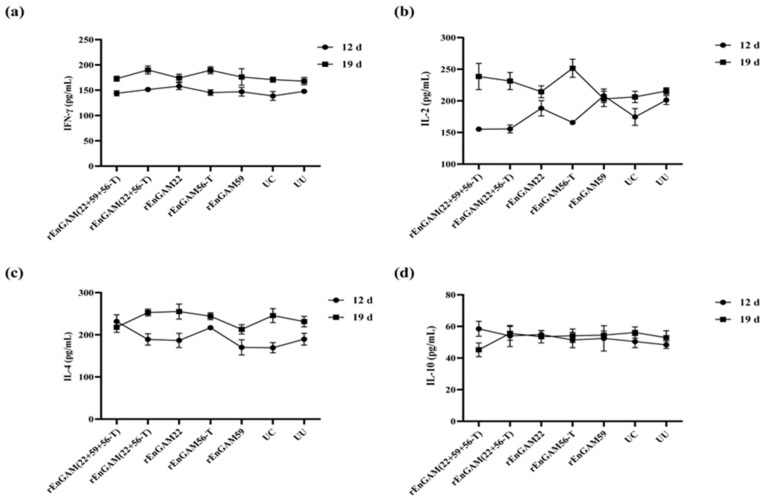
The serum cytokine level responses to single-protein or multi-protein immunization. IFN-γ (**a**), IL-2 (**b**), IL-4 (**c**), and IL-10 (**d**) were detected by ELISA at 7 days following primary and secondary immunization. After the second immunization, no statistically significant differences were observed among the groups. However, the levels of IFN-γ and IL-2 showed an upward trend across all groups. IL-4 levels increased with the age of the chickens in all groups, while IL-10 levels remained relatively stable throughout the time points. Each bar represents the mean ± SEM (*n* = 5).

No statistically significant differences in cytokine expression levels were observed among the groups in response to SCL stimulation. The highest level of IFN-γ (Figure 7a) was detected in the rEnGAM (22 + 56-T) group (190.01 ± 5.07 pg/mL), followed closely by the rEnGAM56-T group (189.44 ± 7.88 pg/mL). Regarding IL-2 levels (Figure 7b), the rEnGAM56-T group (251.62 ± 14.43 pg/mL) exhibited the highest level, followed by the rEnGAM (22 + 59 + 56-T) group (238.50 ± 6.83 pg/mL). The highest IL-4 level (Figure 7c) was observed in the rEnGAM22 group (255.23 ± 17.98 pg/mL). In contrast, IL-10 levels (Figure 7d) did not show an increasing trend with the number of immunizations.

Based on a comprehensive evaluation, the combined immunization group demonstrated enhanced humoral and cellular immune responses compared to the individual immunization groups. According to the overall ACI analysis, the rEnGAM (22 + 59 + 56-T) immunization group was determined to have the most effective immunological response.

## 4. Discussion

The gametocyte proteins serve as a precursor to the oocyst wall protein [20,21]. Several gametocyte antigens have been studied for immunization targets against various parasites, including *E. maxima* [22,23], *E. tenella* [8,13], and *Plasmodium falciparum* [24]. However, few reports have focused on gametocyte antigens from *E. necatrix*. In our previous work, we successfully cloned and expressed the *E. necatrix* gametocyte antigens EnGAM22 and EnGAM59. Subsequent studies demonstrated that the rEnGAM22 and rEnGAM59 provide protective immunity against *E. necatrix* infection, with optimal immunizing doses determined to be 50 μg and 100 μg, respectively.

Belli et al. [11,12,25] observed that EmGAM56 and EmGAM82 were proteolytically processed into small tyrosine-rich peptides with molecular weights of 8, 10, 12, and 31 kDa during oocyst wall formation. Comparable tyrosine-rich proteins originating from gametocyte proteins have also been identified in *E. tenella* and *Eimeria acervuline* [26]. In *E. maxima*, two gametocyte proteins, EmGAM82 (characterized by a high content of tyrosine and serine) and EmGAM56 (enriched in both tyrosine-serine and proline-methionine motifs), are localized within the WFBII and contribute to the formation of the inner oocyst wall. Similarly, EnGAM59, a structural homolog of EmGAM56 identified in *E. necatrix*, contains tyrosine-serine- and proline-methionine-rich domains, enabling its association with WFBII and playing a critical role in the formation of the inner oocyst wall. In contrast, EnGAM22, which is rich in proline and histidine residues, localizes to WFBI and is associated with the outer oocyst wall in *E. necatrix*. In the present study, the full-length EnGAM56, which includes both tyrosine-rich and proline-methionine-rich regions, exhibits an amino acid composition similar to that of EtGAM56 and EmGAM56 and was found to localize to both WFBII and the inner oocyst wall. These findings suggest distinct molecular pathways underlying the formation of the outer and inner oocyst walls.

Wallach et al. [7] found that maternal antibodies produced by breeder hens immunized with *E. maxima* gametocyte antigens confer partial protective immunity to offspring against both homologous and heterologous *Eimeria* infections. Building upon these findings, our previous studies have further confirmed that gametocyte protein 56 (GAM56) represents a promising candidate antigen for the development of vaccines against coccidial infections [27,28,29]. In this study, our results revealed that both rEnGAM56-F and rEnGAM56-T could provide effective immune protection against *E. necatrix* infection, with the protective effects being stronger with rEnGAM56-T. In particular, the group rEnGAM56-T-L achieved the best performance in RWG (84.09%), oocyst production (66.20%), and ACI (158.59), followed by the group rEnGAM56-F-L. The oocyst reduction rate in the rEnGAM56-T-M group reached 58.2%, and the ACI index was 152.79.

The full-length rEnGAM56 protein exhibited lower immunoprotective efficacy compared to its truncated counterpart, aligning with findings reported in previous studies [30,31]. This reduced efficacy may be attributed to the masking of critical antigenic epitopes by other domains within the full-length protein, thereby diminishing the host’s immune recognition and response. Moreover, the structural complexity of the full-length protein increases the likelihood of misfolding, adversely affecting both antigen presentation and protein stability. In contrast, the truncated form facilitates better exposure of key antigenic epitopes, reduces the potential for misfolding, and enhances overall structural stability, collectively contributing to a more robust immune response. Additionally, truncation may eliminate immunosuppressive epitopes present in the full-length protein, further improving its immunogenicity. In terms of antibody levels, group rEnGAM56-T-L showed lower antibody levels following the second immunization compared to group rEnGAM56-T-M. This phenomenon may be attributed to the typical temporal lag in antibody production, wherein antibody titers do not reach their peak until a defined period following infection or immunization. During this interval, despite relatively low circulating antibody levels, other branches of the immune system, such as cellular immunity, may have been activated and effectively suppressed the *E. necatrix* infection. This early immunity may account for the higher ACI observed in the rEnGAM56-T-L group.

Coccidiosis is a complex parasitic disease, and relying on a single antigen presents challenges in effectively targeting its diverse antigenic epitopes and multiple developmental stages. Therefore, incorporating a combination of antigens or multi-epitope vaccine strategies may be necessary to enhance protective efficacy and broaden the immune response spectrum. In an earlier study, immunization with 50 μg of rEnGAM22 resulted in a 15.82% reduction in oocyst shedding. Li [32] reported a 39.55% reduction in oocyst output and an 85.31% relative weight gain in birds immunized with 200 μg of rEnGAM59. Based on these findings, the immunization dose for rEnGAM22 in the present study was set at 50 μg. Although 200 μg was previously identified as the optimal dose for rEtGAM59, this was reduced to 100 μg in the combined immunization strategy to minimize the total protein load per bird, thereby ensuring safety and avoiding potential immunity overstimulation. Remarkably, in the present study, the rEnGAM (22 + 59 + 56-T) and rEnGAM (22 + 56-T) groups achieved RWG values of 96.16% and 91.56%, respectively, both significantly higher than those observed in the UC group. The rEnGAM (22 + 59 + 56-T) group exhibited the most favorable overall performance, with the lowest lesion score (0.30 ± 0.08), the highest antibody level (OD_450_ = 1.35 ± 0.05), a 29.84% reduction in oocyst shedding, and the highest anticoccidial index (ACI) of 173.16, collectively indicating a robust and highly effective anticoccidial immune response.

*E. necatrix* is an intracellular parasite that mainly induces the host cellular immune response. Cytokines are a class of immune factors secreted by immune cells and play an important role in the production and regulation of cellular immune response. Interferon γ (IFN-γ) and Interleukin-2 (IL-2) are classified as Th1 cytokines that could enhance the cellular immune response and mediate delayed hypersensitivity; Interleukin-4 (IL-4) and Interleukin-10 (IL-10) are typical Th2 cytokines, which mainly play a role in enhancing the humoral and allergic immune response [33]. In this study, no significant differences were observed in the levels of IFN-γ, IL-2, IL-4, or IL-10 across all groups. However, IFN-γ and IL-2 levels exhibited an upward trend following secondary immunization. IL-4 levels increased with the age of the chickens in all groups, while IL-10 levels showed no variation at any time point. Subunit vaccines primarily elicit a humoral immune response through specific antigen fragments, while their ability to stimulate cellular immunity often relies on the careful selection of antigens and the incorporation of appropriate adjuvants [6]. Although elevated antibody levels serve as a favorable indicator of vaccine-induced immunity, an insufficient cellular immune response remains a critical limiting factor in achieving full protection. Therefore, balancing humoral and cellular immunity is essential for eliciting comprehensive and long-lasting protection. Optimizing vaccine strategies to address this imbalance is imperative. Future research should focus on incorporating immune adjuvants capable of selectively enhancing Th1-type responses to bolster cellular immunity. Moreover, the development of multi-antigen combination immunization approaches may synergistically stimulate both humoral and cellular arms of the immune system, thereby improving overall immunoprotective efficacy.

## 5. Conclusions

In this study, we successfully cloned, expressed, and characterized the full-length En*gam56* gene from *E. necatrix*, revealing a 1407 bp open reading frame encoding a 468-amino-acid protein with distinct structural and antigenic features. rEnGAM56-F was efficiently expressed in *E. coli*, purified via Ni-NTA affinity chromatography, and demonstrated strong immunoreactivity in Western blot assays with anti-6 × HIS monoclonal antibody, anti-rEnGAM56-F polyclonal antibody, and convalescent serum from chickens infected with *E. necatrix*. Immunofluorescence analysis further confirmed its localization to WFB2 in gametocytes and the oocyst wall, implying a functional role in parasite wall biosynthesis. These results suggest that EnGAM56 is a structurally conserved, immunogenic gametocyte antigen with potential relevance in host–parasite interactions and vaccine development.

Vaccination trials demonstrated that rEnGAM56, particularly the truncated version (rEnGAM56-T), conferred partial protection against *E. necatrix* infection, as evidenced by improved body weight gain, reduced lesion scores, and enhanced anticoccidial index (ACI). Moreover, the multivalent antigen combination including rEnGAM22, rEnGAM59, and rEnGAM56-T elicited the most robust protective immunity, achieving an ACI of 173.16 and significantly elevated IgY levels. These findings underscore the potential of EnGAM56, especially when incorporated into multi-antigen formulations, as a promising component of next-generation subunit vaccines against avian coccidiosis caused by *E. necatrix*.

## Figures and Tables

**Table 1:** List of each primer sequence.

Primer Name	Primer Sequence 5′→3′
En*gam56*-F1-F	ATGACTCGCCTCAGCCTC
En*gam56*-F1-R	GGTCGTTGTCGTGGTGCT
En*gam56*-F2-F	CATATGGTGGAGAACACGGTGCAC
En*gam56*-F2-R	CTCGAGTTAGTACCAGCTGGAGGAGTA
En*gam56*-F3-F	CACTGCTGTTGAGAAGGAGGAAACTGCC
En*gam56*-F3-R	TTACGGAGGAGTGGGGACGAAGCTGAAG

**Table 2:** Protective efficacy of rEnGAM56-F or rEnGAM56-T protein vaccination on *E. necatrix* challenge.

Groups	Average Body Weight Gains 1 (g)	Average Body Weight Gains 2 (g)	Relative Weight Gain	Lesion Scores	Oocyst Shedding Per Bird (× 10^7^)	Oocyst Reduction Rate (%)	Anti-Coccidial Index (ACI)
rEnGAM56-F-H	252.99 ± 4.42 ^a^	184.06 ± 16.99 ^a^	71.67%	2.00 ± 0.52 ^bc^	2.64	34.33%	131.67
rEnGAM56-F-M	253.66 ± 3.70 ^a^	208.93 ± 14.55 ^ab^	81.35%	1.85 ± 0.97 ^bc^	3.28	18.59%	122.85
rEnGAM56-F-L	258.53 ± 1.83 ^a^	214.38 ± 13.10 ^ab^	83.47%	1.70 ± 0.75 ^bc^	2.02	49.85%	146.47
rEnGAM56-T-H	258.95 ± 4.09 ^a^	193.63 ± 19.12 ^a^	75.39%	2.35 ± 0.78 ^bc^	1.72	57.16%	141.89
rEnGAM56-T-M	256.01 ± 5.08 ^a^	194.66 ± 15.50 ^a^	75.79%	1.30 ± 1.09 ^b^	1.68	58.20%	152.79
rEnGAM56-T-L	256.72 ± 6.23 ^a^	215.97 ± 20.72 ^ab^	84.09%	1.55 ± 0.72 ^bc^	1.36	66.20%	158.59
UC	257.71 ± 3.36 ^a^	182.57 ± 16.80 ^a^	71.09%	2.50 ± 1.08 ^c^	4.02	0.00%	106.09
UU	258.16 ± 2.48 ^a^	256.83 ± 4.35 ^b^	-	0.00 ± 0.00 ^a^	0.00	0.00%	-

Note: a–c values with different letters in the same column are significantly different according to the ANOVA Duncan test (*p* < 0.05).

**Table 3:** Protective efficacy of single-protein or multiple-protein vaccination on *E. necatrix* challenge.

Groups	Average Body Weight Gains 1 (g)	Average Body Weight Gains 2 (g)	Relative Weight Gain	Lesion Scores	Oocyst Shedding Per Bird (× 10^6^)	Oocyst Reduction Rate (%)	Anti-Coccidial Index (ACI)
rEnGAM (22 + 59 + 56-T)	288.79 ± 5.97 ^a^	279.03 ± 5.04 ^b^	96.16%	0.30 ± 0.08 ^a^	1.97	29.84%	173.16
rEnGAM (22 + 56-T)	290.56 ± 5.04 ^a^	265.68 ± 14.66 ^b^	91.56%	1.00 ± 0.18 ^ab^	1.82	35.04%	161.56
rEnGAM22	292.31 ± 4.25 ^a^	258.26 ± 12.35 ^ab^	89.00%	0.70 ± 0.11 ^ab^	1.67	40.38%	162.00
rEnGAM56-T	290.76 ± 5.15 ^a^	239.2 ± 11.11 ^a^	82.43%	1.40 ± 0.24 ^bc^	2.45	12.82%	128.43
rEnGAM59	289.52 ± 4.58 ^a^	253.53 ± 13.10 ^ab^	87.37%	1.70 ± 0.22 ^bc^	2.60	7.48%	130.37
UC	290.14 ± 3.93 ^a^	231.78 ± 13.62 ^a^	79.88%	2.10 ± 0.25 ^c^	2.81	0.00%	118.88
UU	290.50 ± 2.05 ^a^	290.17 ± 9.43 ^b^	-	0.00 ± 0.00 ^a^	0.00	0.00%	-

Note: a–c values with different letters in the same column are significantly different according to the ANOVA Duncan test (*p* < 0.05).

## Data Availability

The raw data supporting the conclusions of this article will be made available by the authors on request.
